# *Enterobacteriaceae* Isolated from the River Danube: Antibiotic Resistances, with a Focus on the Presence of ESBL and Carbapenemases

**DOI:** 10.1371/journal.pone.0165820

**Published:** 2016-11-03

**Authors:** Clemens Kittinger, Michaela Lipp, Bettina Folli, Alexander Kirschner, Rita Baumert, Herbert Galler, Andrea J. Grisold, Josefa Luxner, Melanie Weissenbacher, Andreas H. Farnleitner, Gernot Zarfel

**Affiliations:** 1 Institute of Hygiene, Microbiology and Environmental Medicine, Medical University Graz, Graz, Austria; 2 Institute for Hygiene and Applied Immunology, Water Hygiene, Medical University of Vienna, Vienna, Austria; 3 Interuniversity Cooperation Centre for Water and Health; 4 Institute of Chemical Engineering, Research Group Environmental Microbiology and Molecular Ecology, Vienna University of Technology, Vienna, Austria; UNITED STATES

## Abstract

In a clinical setting it seems to be normal these days that a relevant proportion or even the majority of different bacterial species has already one or more acquired antibiotic resistances. Unfortunately, the overuse of antibiotics for livestock breeding and medicine has also altered the wild-type resistance profiles of many bacterial species in different environmental settings. As a matter of fact, getting in contact with resistant bacteria is no longer restricted to hospitals. Beside food and food production, the aquatic environment might also play an important role as reservoir and carrier. The aim of this study was the assessment of the resistance patterns of *Escherichia coli* and *Klebsiella* spp. out of surface water without prior enrichment and under non-selective culture conditions (for antibiotic resistance). In addition, the presence of clinically important extended spectrum beta lactamase (ESBL) and carbapenmase harboring *Enterobacteriaceae* should be investigated. During Joint Danube Survey 3 (2013), water samples were taken over the total course of the River Danube. Resistance testing was performed for 21 different antibiotics. Samples were additionally screened for ESBL or carbapenmase harboring *Enterobacteriaceae*. 39% of all isolated *Escherichia coli* and 15% of all *Klebsiella* spp. from the river Danube had at least one acquired resistance. Resistance was found against all tested antibiotics except tigecycline. Taking a look on the whole stretch of the River Danube the proportion of multiresistances did not differ significantly. In total, 35 ESBL harboring *Enterobacteriaceae*, 17 *Escherichia coli*, 13 *Klebsiella pneumoniae* and five *Enterobacter* spp. were isolated. One *Klebsiella pneumoniae* harboring NMD-1 carbapenmases and two *Enterobacteriaceae* with KPC-2 could be identified. Human generated antibiotic resistance is very common in *E*. *coli* and *Klebsiella* spp. in the River Danube. Even isolates with resistance patterns normally associated with intensive care units are present.

## Introduction

We are currently observing the spread of a rising number of anthropogenic antibiotic resistant bacteria (ARB) outside the clinical setting. This is an alarming trend, contributing to the postulated decline of the antibiotic era [1–3]. Especially surface waters seem to play a key role in this spread, as they serve both as habitats and as transport systems for microorganisms [4]. Contrary to clinical settings, where the distribution of resistant bacteria is well-documented [3,5], distribution and evidence of non-wild-type resistant pathogens in the environment are hardly based on qualitative data.

Antibiotics and ARB stem from many different sources like hospital effluents, communities, industry and farming and are flushed into surface waters. This leads to an emerging number of ARB in the environment [6–8]. The ability of resistant bacteria to survive in the aquatic environment and the transfer of resistance genes are not clearly understood. Besides genetic background of the strains and mobility of resistance genes the presence of antibiotics, their degradation products or other substances i.e. metals can influence the stability of the resistance [4,6,7,9]. Antibiotic resistant gram negative bacilli (e.g. *Enterobacteriaceae*, *Pseudomonadales*) are favored, as many species are native inhabitants of water environments and they are capable of high trans-species genetic exchanges [4,10]. So today surface waters may not only serve as reservoirs for resistance genes but also as a “market place” where susceptible strains (especially in the presence of antibiotics from waste water) can acquire new resistances [6–8,11].

Worldwide research document the occurrence and increasing presence of nearly all clinically relevant resistance mechanisms in the *Enterobacteriaceae* family, in all kinds of surface waters from waste to drinking water, in rivers, lakes and in the ocean [12–16].

Especially extended spectrum beta-lactamases (ESBL) producing bacteria have become omnipresent in the last decade. They emerge within clinical settings, human communities and animals (wild life, companion animals and livestock) [17,18]. One reason for the increase of ESBL within population and in animals is the overuse of antimicrobials in veterinary medicine. This leads to the occurrence of ARB in the animals itself and a contamination of the foodstuff of animal origin. The spread of the ARB loaded manure then contaminates soil or surface waters [19,20].

**Fig 1:**
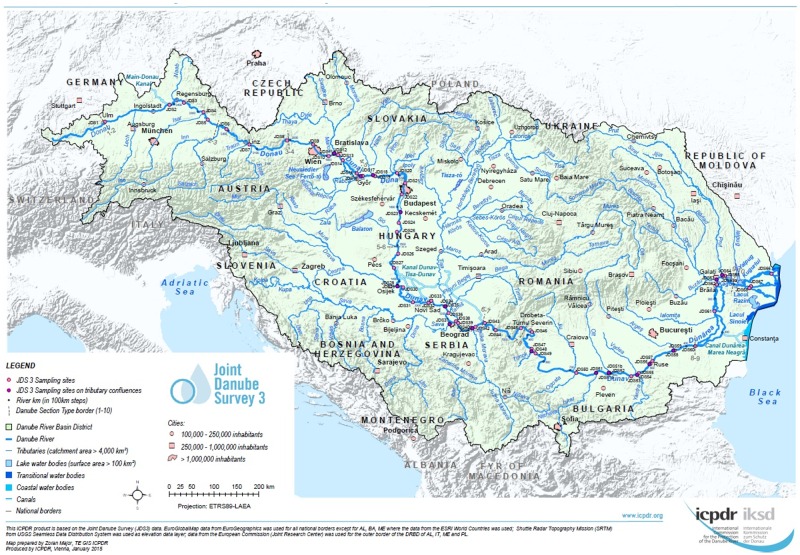
JDS 3 overview map. Overview of the Joint Danube Survey 3 sampling points (JDS3) along the River Danube. Reprinted from Joint Danube Survey Webpage (http://www.icpdr.org/main/activities-projects/jds3) under a CC BY license, with permission from the ICPDR (International Commission for the Protection of the Danube River), original copyright 2013.

The aim of the study was to evaluate the resistance profiles of *Escherichia coli* and *Klebsiella* spp. isolated at selected sites along the whole course of the River Danube. We took the opportunity of the Joint Danube Survey 3 (JDS3), the world's biggest river research expedition of its kind in 2013, to analyze samples originating from different sampling points along the whole length of the River Danube. The isolates were collected without any antibiotic pressures or pre-enrichment to provide a mostly unbiased picture of the antibiotic resistance of these clinically highly important micro-organisms in the River Danube. A parallel screening on ESBL and carbapenemases producing *Enterobacteriaceae* in the River Danube water was conducted (Fig 1).

## Materials and Methods

### Sample collection

All samples were taken from a research vessel during the Joint Danube Survey 2013 (JDS3), organized by the International Commission for the Protection of the Danube River (ICPDR), Vienna. ICPDR has got the permission of all Danube countries for taking samples along the whole Danube River. The ICPDR is a transnational body, which has been established to implement the Danube River Protection Convention. All Danube countries are member states of the ICPDR on the base of the “Convention on Cooperation for the Protection and Sustainable use of the Danube River (Danube River Protection Convention).

Between Aug. 12^th^ and Sep. 26^th^, 2013, water samples from 68 sampling sites along the River Danube, starting at JDS1 (Böfinger Halde, DE), and from 12 tributaries were collected for microbiological investigation. For each sampling site, samples were taken at three sampling points (left, middle, right), in sterile 1 L glass flasks from 30 cm below the river surface. From each flask duplicate volumes of 45 ml river water were filled into sterile non-toxic 50 ml plastic vials (Techno Plastic Products AG, TPP, Switzerland), containing 5 ml glycerine (final conc. 10% v/v) [21]. The vials were completely mixed by hand and immediately stored at -20°C on board of the cruise ship until analysis in the laboratory. After transfer to the laboratory (beginning of October 2013) the samples were stored at -80°C. Fourteen critical sampling sites, mostly downstream of large cities were chosen for investigation (Table 1). In order to facilitate a better interpretation of the data, the sampling sites were grouped in three stretches: upper-, middle- and down-stretches (Table 1). The upper stretch included JDS2, JDS3, JDS8, JDS10 and JDS22 (1240 river km), represented by 120 *E*. *coli* and 136 *Klebsella* spp. isolates. The middle stretch included JDS28, JDS36, JDS38 and JDS49 (798 river km), represented by 326 *E*. *coli* and 88 *Klebsella* spp. isolates.

**Table 1:** Sampling sites.

SP	Stretch	Name of SP	River km	Country
JDS2	upper	Kelheim, gauging station	2415	DE
JDS3	upper	Geisling power plant	2354	DE
JDS8	upper	Oberloiben	2008	AT
JDS10	upper	Wildungsmauer (Vienna)*	1895	AT
JDS22	upper	ds Budapest*	1632	HU
JDS28	middle	us Drava*	1384	HR/RS
JDS36	middle	ds Tisa / us Sava	1200	RS
JDS38	middle	us Pancevo (Belgrade)*	1159	RS
JDS49	middle	Pristol / Novo Salo	834	RO/BG
JDS57	down	ds Ruse*	488	RO/BG
JDS59	down	ds Arges (Bucharest)*	429	RO/BG
JDS63	down	Siret	154	RO
JDS67	down	Sulina Arm	26	RO
JDS68	down	St.Gheorge Arm	104	RO

JDS3 sampling sites chosen for isolation and their assignment to the upper-, middle- or downstream stretches (SP = sampling point, us = upstream, ds = downstream). Country codes: Germany, DE; Austria, AT; Hungary, HU; Croatia, HR; Serbia, RS; Romania, RO; Bulgaria, BG;

* represent sites close to cities.

The down stretch included JDS57, JDS59, JDS63, JDS67 and JDS68 (808 river km), represented by 183 *E*. *coli* and 95 *Klebsella* spp. isolates.

Each of the defined stretches included two sampling points after waste water treatment plants (WWTP) supporting large cities (Table 1, marked with *), and two sampling sites with not such an influence. In addition starting sampling site at Kehlheim (JDS2) and final sampling site at the River Danube delta St. Gheorge Arm (JDS68) were included also.

### Isolation of bacteria

Isolation of *E*. *coli* and *Klesbsiella* spp. without resistance selection

The frozen samples were thawed and plated afterwards in 0.5 ml portions on different (selective) Agars. For each sampling point ten Agar plates of each type were used. For isolation of *E*. *coli* and *Klebsiella* spp. Endo Agar, Xylose Lysine Desoxychelat Agar (XLD Agar) and Chromocult Coliform Agar (CCA), (all Merck, Austria) were used. Growth conditions were 37 ± 1°C for 18–24 h. All colonies that matched manufacturers’ requirements were transferred to Blood Agar and Endo Agar (24 h, 37°C) to retrieve pure cultures. Species were identified with mass spectrometry VITEK^®^ MS (bioMérieux Austria GmbH, Vienna, Austria). These isolates were used for determination of wild-type, resistant and multiresistant proportion of River Danube *E*. *coli* and *Klebsiella* spp.

Isolation of ESBL and/or Carbapenemases harboring *Enterobacteriaceae*

The frozen samples were thawed and plated afterwards in 0.5 ml portions on different ChromeID^®^ Agars. For each sampling point ten Agar plates of each type were used. ChromID^®^ ESBL (bioMérieux Austria GmbH, Vienna, Austria) and chromID^®^ CARBA (bioMérieux Austria GmbH, Vienna, Austria) were used for screening for ESBL and for carbapenemase-producing *Enterobacteriaceae*. ChromID^®^ Agar plates were incubated for 24 h at 37°C. Colonies were assessed as described in the manufacturer´s manual. For pure cultures, colonies growing on chromID^®^ Agar were transferred to blood Agar and Endo Agar (24 h, 37°C) and identified with MALDI-TOF Vitek^®^ MS. These isolates were not included in the calculation of wild-type, resistant and multiresistant proportion of River Danube *E*. *coli* and *Klebsiella* spp.

### Susceptibility testing

For all identified *Enterobacteriaceae* susceptibility testing was performed as recommended by the European Committee on Antimicrobial Susceptibility testing (EUCAST) [22]. If no EUCAST criteria were available (tetracycline, chloramphenicol and nalidixic acid), testings were carried out according to the Clinical Laboratory Standards Institute (CLSI) [23]. Interpretation of zone diameters was done according to EUCAST or CLSI.

The following antibiotics (21) were used: ampicillin (10 μg), amoxicillin/clavulanic acid (20 μg/10 μg), piperacillin/tazobactam (100 μg/10 μg), cefalexin (30 μg), cefuroxime (30 μg), cefoxitin (30 μg), cefotaxime (5 μg), ceftazidime (10 μg), cefepime (30 μg), imipenem (10 μg), meropenem (10 μg), amikacine (30 μg), gentamicin (10 μg), trimethoprim/sulfamethoxazole (1.25 μg/23.75 μg), ciprofloxacin (5 μg), moxifloxacin (5 μg), tigecyclin (15 μg), tetracycline (30 μg), nalidixic acid (30 μg) chloramphenicol (30 μg) and colistin (10 μg) (Becton Dickinson and Company, Sparks, MD, USA, BD BBL^™^). Sensi-DiscTM paper discs (BD) were used.

According to EUCAST test criteria for disc diffusion, are only available for *E*. *coli*. Susceptibility for all other *Enterobacteriaceae* has to be determined with Etest^®^. Etest for tigecyclin was carried out and interpreted according to EUCAST guidelines.

To determine (clinical) resistance to colistin protocols of Gales et al. and Boyen et al. were used [24,25].

*Escherichia coli* ATCC 25922 and *Pseudomonas aeruginosa* ATCC 27853 were used as control strains in all performed tests.

Phenotypically conformation of ESBL and Carbapenmases

The minimum inhibitory concentrations (MICs) for imipenem and meropenem were tested with Etest^®^ (bioMérieux Austria GmbH, Vienna, Austria). Expression of carbapenmases was confirmed with modified hodge test [26]. ESBL-positive *E*. *coli* and *Klebsiella* spp. were confirmed with double disc tests (CLSI) (30 μg ceftazidime, 30 μg cefepime, ceftazidime-clavulanic acid 30/10 μg, cefepime-clavulanic acid 30/10 μg; bioMérieux Austria GmbH, Vienna, Austria). ESBL-positive *Enterobacter* spp. and *Citrobacter* spp. were confirmed with modified double-disc test [27]. *Escherichia coli* ATCC 25922 and *Pseudomonas aeruginosa* ATCC 27853 were used as control strains in all performed tests. Isolates that revealed ESBL and/or Carbapenemase phenotype were tested for their genetic background.

### Determination of ESBL and Carbapenemase genes

PCR detection and gene identification were performed for five different β-lactamase gene families, *bla*_CTX-M-1group_, *bla*_CTX-M-2group_, *bla*_CTX-M-9group_, *bla*_TEM_, and *bla*_SHV_. DNA was extracted by boiling of one colony suspended in 50 μl double-deionized water (95°C for 10 min.) After centrifugation for 1 min at 13000 rpm (Centriduge 5415 R, Eppendorf) supernatant was used for PCR—reaction. PCR and sequencing procedures were performed as described previously [28,29]. Standard PCR protocols and conditions were modified in the following way: initial denaturation at 94°C for 5 min; 35 cycles at 95°C for 30 sec, 52°C for 45 sec, and 72°C for 60 sec; and final incubation for 10 min at 72°C using Taq DNA polymerase and dNTPs from QIAGEN (Hilden, Germany). This was done for all *Enterobacteriaceae* isolates that revealed an ESBL-positive phenotype or were recovered from chromID^®^ CARBA plates. Isolates showing resistance to at least one of the tested carbapenems were screened with a Checkpoint MDR 103 kit (Check-Points, Wageningen, The Netherlands) according to the protocol http://www.check-points.com/support/manuals/. Detected carbapenemase genes (*bla*_NDM,_ *bla*_KPC_) were characterized by sequencing as described previously [30]. DNA extraction was done as described above for ESBL genes. Standard PCR protocols: initial denaturation at 94°C for 5 min; 35 cycles at 95°C for 30 sec, 52°C for 45 sec, and 72°C for 60 sec; and final incubation for 10 min at 72°C using Taq DNA polymerase and dNTPs from QIAGEN (Hilden, Germany).

### Multilocus sequence typing (MLST)

MLST for *E*. *coli* was done according to the MLST Databases at University of Warwick (http://mlst.warwick.ac.uk/mlst/dbs/Ecoli/) [31] and for *Klebsiella pneumonie* according to the Institute Pasteur MLST (http://bigsdb.web.pasteur.fr/klebsiella/klebsiella.html) [32,33].

MLST of *Enterobacter cloacae* was done according to the *Enterobacter cloacae* MLST website (http://pubmlst.org/ecloacae/) developed by Keith Jolley and sited at the University of Oxford [31]. The development of this site has been funded by the Wellcome Trust.

### Statistical analyses

Statistical analyses were carried out using R^®^ Version 3.21, a free software environment for statistical computing and graphics (www.r-project.org). Group specific proportions were tested on their equality by a two-sided binomial test. *P*values below 0.05 were assessed as significant.

## Results

### Resistance pattern of *E*. *coli* and *Klebsiella* spp.

In total, 629 *E*. *coli* and 319 *Klebsiella* spp. (238 *Klebsiella pneumoniae* and 81 *Klebsiella oxytoca*) were isolated under non-selective conditions (according antibiotic resistance).The presence of acquired resistances in the total population was tested for their susceptibility to 21 antibiotics, including clinically relevant antibiotics as well as antibiotics commonly used in farming (e.g. tetracycline).

61.21% (385 isolates) of all *E*. *coli* isolates and 84.01% (268 isolates) of *Klebsiella* spp. isolates did not show acquired resistance to any of the tested antibiotics (*Klebsiella* spp. are set as naturally resistant to ampicillin). 61 *E*. *coli* isolates (9.70%) and 7 *Klebsiella* spp. (2.19%) were identified as multiresistant (acquired resistance to three or more antibiotic classes tested). *E*. *coli* isolates were resistant to up to 14 of 21 tested antibiotics and six of seven tested classes, *Klebsiella* spp. were resistant to up to 12 of 20 antibiotics and five of seven classes. Four isolates (two *E*. *coli* and two *Klebsiella pneumoniae*) were tested as positive as regards harboring ESBL genes and were analyzed together with ESBL positive isolates from ChromID ESBL and ChromID CARBA Agar plates. All isolates were susceptible to meropenem, imipenem, amikacine and tigecycline. Additionally all *E*. *coli* isolates were susceptible to piperacillin/tazobactam and colistin, while two *Klebsella* spp. isolates were resistant to these antibiotics. The most common resistance in both tested species was tetracycline with 24.01% of all isolated *E*. *coli* and 8.46% of all *Klebsiella* spp. Resistance to ampicillin (21.94%) was second common in *E*. *coli* isolates, followed by nalidixic acid (10.97%), trimethoprim/sulfamethoxazole (10.17%) and amoxicillin/clavulanic acid (5.88%). All other antibiotics revealed resistance only in less than 5% of the isolates. *Klebsiella* spp. isolates also revealed resistance to tetracycline (8.46%), amoxicillin/clavulanic acid (6.03%) and nalidixic acid (5.02%) with resistance proportions higher than 5% (Table 2).

**Table 2:** Proportion of antibiotic resistance.

	*E*. *coli* up	*E*. *coli* middle	*E*. *coli* down	*E*. *coli* all	*Klebs*. spp up	*Klebs*. spp middle	*Klebs*. spp down	*Klebs*. spp all
total	121	326	183	629	136	88	95	319
ampicillin	40 (33.33%)	66 (20.18%)	31 (16.94%)	137 (21.78%)	136 (100%)	88 (100%)	95 (100%)	319 (100%)
^a^amox./clavul.	10 (8.33%)	18 (5.50%)	9 (4.92%)	37 (5.88%)	12 (8.82%)	1 (1.14%)	4 (4.21%)	17 (5.33%)
cefalexin	6 (5.00%)	7 (2.14%)	4 (2.19%)	17 (2.70%)	7 (5.15%)	0 (0.00%)	1 (1.05%)	8 (2.51%)
cefuroxime	0 (0.00%)	5 (1.53%)	2 (1.09%)	7 (1.11%)	2 (1.47%)	1 (1.14%)	1 (1.05%)	4 (1.25%)
cefoxitin	6 (5.00%)	3 (0.92%)	2 (1.09%)	11 (1.75%)	12 (8.82%)	1 (1.14%)	0 (0.00%)	9 (2.82%)
cefotaxime	0 (0.00%)	3 (0.92%)	2 (1.09%)	5 (0.79%)	2 (1.47%)	0 (0.00%)	1 (1.05%)	3 (0.94%)
gentamicin	6 (5.00%)	9 (2.75%)	1 (0.55%)	16 (2.54%)	2 (1.47%)	1 (1.14%)	1 (1.05%)	4 (1.25%)
^b^pip./taz.	0 (0.00%)	0 (0.00%)	0 (0.00%)	0 (0.00%)	1 (0.74%)	0 (0.00%)	1 (1.05%)	2 (0.63%)
moxifloxacin	8 (6.67%)	16 (4.89%)	5 (2.73%)	29 (4.61%)	2 (1.47%)	1 (1.14%)	3 (3.16%)	6 (1.88%)
ciprofloxacin	5 (4.17%)	16 (4.89%)	4 (2.19%)	25 (3.97%)	1 (0.74%)	1 (1.14%)	1 (1.05%)	3 (0.94%)
^c^SXT	6 (5.00%)	44 (13.46%)	14 (7.65%)	64 (10.17%)	4 (2.94%)	1 (1.14%)	2 (2.11%)	7 (2.19%)
meropenem	0 (0.00%)	0 (0.00%)	0 (0.00%)	0 (0.00%)	0 (0.00%)	0 (0.00%)	0 (0.00%)	0 (0.00%)
amikacine	0 (0.00%)	0 (0.00%)	0 (0.00%)	0 (0.00%)	0 (0.00%)	0 (0.00%)	0 (0.00%)	0 (0.00%)
imipenem	0 (0.00%)	0 (0.00%)	0 (0.00%)	0 (0.00%)	0 (0.00%)	0 (0.00%)	0 (0.00%)	0 (0.00%)
cefepime	0 (0.00%)	2 (0.61%)	0 (0.00%)	2 (0.32%)	1 (0.74%)	0 (0.00%)	1 (1.05%)	2 (0.63%)
ceftazidime	0 (0.00%)	4 (1.22%)	2 (1.09%)	6 (0.95%)	1 (0.74%)	0 (0.00%)	2 (2.11%)	3 (0.94%)
tigecycline	0 (0.00%)	0 (0.00%)	0 (0.00%)	0 (0.00%)	0 (0.00%)	0 (0.00%)	0 (0.00%)	0 (0.00%)
tetracycline	11 (9.17%)	88 (26.91%)	52 (28.42%)	151 (24.01%)	9 (6.62%)	3 (3.41%)	15 (15.79%)	27 (8.46%)
chloramphenicol	4 (3.33%)	18 (5.50%)	9 (4.92%)	31 (4.93%)	6 (4.41%)	2 (2.27%)	2 (2.11%)	10 (3.13%)
nalidixic acid	14 (11.67%)	44 (13.46%)	11 (6.01%)	69 (10.97%)	5 (3.68%)	3 (3.41%)	8 (8.42%)	16 (5.02%)
colistin	0 (0.00%)	0 (0.00%)	0 (0.00%)	0 (0.00%)	1 (0.74%)	1 (1.14%)	0 (0.00%)	2 (0.63%)

Numbers (and proportion) of *E*. *coli* and *Klebsiella* spp. with resistance to tested antibiotics.

^a^ amoxicillin/clavulanic acid, amox./clavul.;

^b^ piperacillin/tazobactam, pip./taz.;

^c^ trimethoprim/sulfamethoxazole, SXT

The upper stretch had the highest proportion of isolates resistant to ampicillin (*E*. *coli*) with the highest percentage of 33.33% against a single antibiotic in this study. In these isolates resistance to amoxicillin/clavulanic, cephalexin and cefoxitin was also commonly found. Isolates that revealed resistance to higher generation cephalosporins occurred only sporadically (five or less isolates per stretch), with the one remark that no *E*. *coli* revealed this resistance in the upstream section. In contrast to the beta-lactam antibiotics, resistance to tetracycline was highest downstream (Table 2).

**Fig 2:**
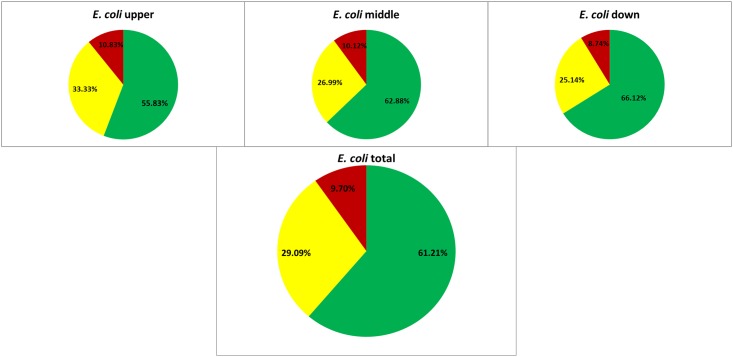
Resistance proportion of *E*. *coli*. Proportion of *E*. *coli* with wild type susceptibility pattern (green), resistance to antibiotics out of one or two tested classes (resistant, yellow) and resistance to antibiotics out of three or more classes (multiresistant, red) their total presence in the river and in the three stretches.

The proportion of multiresistant *E*. *coli* did not change significantly (in comparison upper- to middle stretch: *P*value = 0.9 and middle- to down stretch *P*value = 0.72) over the three stretches with upper- 10.83% (13/120 isolates), middle- 10.12% (33/326 isolates) and downstream 8.74% (16/183 isolates) respectively (Fig 2).

**Fig 3:**
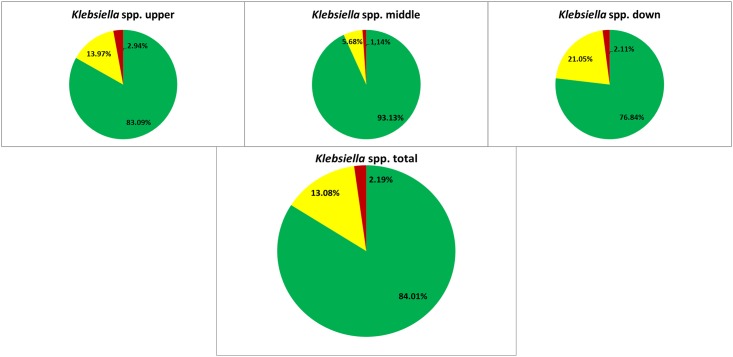
Resistance proportion of *Klebsiella* spp. Proportion of *Klebsiella* spp. with wild type susceptibility pattern (green), resistance to antibiotics out of one or two tested classes (resistant, yellow) and to antibiotics out of three or more classes (multiresistant, red) their total presence in the river and in the three stretches.

The low number of multiresistant bacteria in upper- 2.94%, middle- 1.14% and down stretch 2.11% proportion isolates of *Klebsiella* spp. did not allow for reliable statistic evidence (Fig 3).

### Detection and characterization of ESBL harboring *Enterobacteriaceae*

ESBL and carbapenemases were chosen as examples for clinically important resistance mechanisms. The five ESBL positive isolates which were obtained under non-selective condition from the resistance patterns of *E*. *coli* and *Klebsiella spp*. part were included for detailed analysis. All other isolates except JDS59EB009 that could be obtained from the chromID^™^ CARBA Agar were isolated from ChromeD^™^ ESBL.

In total, 35 ESBL harboring *Enterobacteriaceae*, 17 *E*. *coli*, 13 *Klebsiella pneumoniae* and five *Enterobacter* spp. were isolated. These isolates were obtained from seven of fourteen sampling sites, JDS02 (DE), JDS36 (RS), JDS38 (RS), JDS59 (RO), JDS63 (RO) and JDS68 (RO). In the upper stretch only one isolate (JDS02KL027) could be detected, whereas the majority (22/35) was present in the last four sampling sites (Table 3).

**Table 3:** ESBL and carbapenemase harboring isolates.

Isolation-Nr	Spezies	source country	source rkm	AM	AMC	TZP	CN	CXM	FOX	CTX	CAZ	FEP	MEM	IPM	NA	MXF	CIP	GM	AN	TGC	TE	SXT	C	CL	TEM	SHV	CTX-M-1	CTX-M-9	KPC	NDM	MLST
JDS02KL027	*Klebsiella pneumoniae*	DE	2415	R	R	R	R	R	S	R	R	R	S	S	R	R	R	R	S	S	S	R	S	S	TEM-1	SHV-12	CTX-M-15				ST 15
JDS36EC049	*E*. *coli*	RS	1200	R	S	S	R	R	S	R	S	R	S	S	R	S	S	S	S	S	S	R	S	S	TEM-1		CTX-M-1				ST 1914
JDS38EB037	*Enterobacter cloacae*	RS	1158	R	R	S	R	R	R	R	R	S	S	S	R	S	S	S	S	S	S	S	S	S	TEM-3						ST 505
JDS38EC072	*E*. *coli*	RS	1158	R	R	S	R	R	S	R	R	R	S	S	R	R	R	R	S	S	R	S	S	S	TEM-1		CTX-M-15				ST 131
JDS38EC125	*E*. *coli*	RS	1158	R	S	S	R	R	S	R	S	S	S	S	S	S	S	S	S	S	S	S	S	S			CTX-M-1				ST 48
JDS38EC134	*E*. *coli*	RS	1158	R	S	S	R	R	S	R	S	R	S	S	R	R	R	R	S	S	R	S	S	S	TEM-1		CTX-M-15				ST 131
JDS38EC135	*E*. *coli*	RS	1158	R	S	S	R	R	S	R	R	S	S	S	R	R	R	S	S	S	R	R	S	S			CTX-M-3				ST 205
JDS38EC136	*E*. *coli*	RS	1158	R	S	S	R	R	S	R	R	S	S	S	R	R	R	S	S	S	R	R	S	S				CTX-M-27			ST 131
JDS38EC142	*E*. *coli*	RS	1158	R	R	S	R	R	S	R	R	R	S	S	R	R	R	R	S	S	R	R	R	S	TEM-1		CTX-M-15				ST 5688
JDS38KL007	*Klebsiella pneumoniae*	RS	1158	R	R	S	R	R	S	R	R	S	S	S	R	R	R	R	R	S	R	R	S	S			CTX-M-15				ST 15
JDS38KL009	*Klebsiella pneumoniae*	RS	1158	R	R	R	R	R	R	R	R	R	R	R	R	R	R	R	R	S	R	R	R	S	TEM-1	SHV-1	CTX-M-15			NDM-1	ST 101
JDS38KL027	*Klebsiella pneumoniae*	RS	1158	R	R	R	R	R	S	R	R	R	S	S	R	R	R	R	S	S	S	R	S	S	TEM-1	SHV-12	CTX-M-15				ST 15
JDS38KL045	*Klebsiella pneumoniae*	RS	1158	R	R	R	R	R	R	R	R	R	R	R	R	R	R	R	R	S	S	R	S	S		SHV-11	CTX-M-15		KPC-2		ST 2151
JDS59EB001	*Enterobacter cloacae*	RO/BG	429	R	R	S	R	R	R	R	R	R	S	S	R	R	R	R	S	S	R	R	R	S			CTX-M-15				ST 159
JDS59EB009	*Enterobacter asburiae*	RO/BG	429	R	R	R	R	R	R	R	R	R	R	R	R	S	R	R	S	S	R	R	R	S	TEM-1	SHV-12	CTX-M-1		KPC-2		n.t.
JDS59EB028	*Enterobacter cloacae*	RO/BG	429	R	R	S	R	R	R	R	R	S	S	S	R	R	S	R	S	S	R	R	R	S	TEM-1	SHV-12					ST 145
JDS59EC001	*E*. *coli*	RO/BG	429	R	R	S	R	R	R	R	R	R	S	S	R	R	R	R	S	S	R	S	S	S			CTX-M-15				ST 405
JDS59EC002	*E*. *coli*	RO/BG	429	R	R	S	R	R	R	R	R	R	S	S	R	R	R	S	S	S	S	S	S	S	TEM-1		CTX-M-15				ST 405
JDS59EC003	*E*. *coli*	RO/BG	429	R	R	S	R	R	R	R	S	S	S	S	R	S	S	S	S	S	S	R	R	S	TEM-1	SHV-12					ST 5689
JDS59EC004	*E*. *coli*	RO/BG	429	R	S	S	R	R	S	R	S	R	S	S	R	R	R	S	S	S	R	R	S	S			CTX-M-3				ST 10
JDS59EC005	*E*. *coli*	RO/BG	429	R	S	S	R	R	S	R	R	S	S	S	R	R	R	S	S	S	R	S	S	S	TEM-1		CTX-M-15				ST 3171
JDS59EC006	*E*. *coli*	RO/BG	429	R	R	S	R	R	S	R	R	S	S	S	R	S	S	R	S	S	R	R	S	S	TEM-1		CTX-M-15				ST 69
JDS59EC007	*E*. *coli*	RO/BG	429	R	R	S	R	R	S	R	R	R	S	S	S	S	S	S	S	S	R	R	S	S			CTX-M-15				ST 10
JDS59EC008	*E*. *coli*	RO/BG	429	R	S	S	R	R	S	R	R	R	S	S	R	R	R	R	S	S	R	R	S	S			CTX-M-15				ST 10
JDS59EC009	*E*. *coli*	RO/BG	429	R	R	R	R	R	R	R	S	R	S	S	R	R	R	S	S	S	R	R	S	S	TEM-1		CTX-M-1				ST 617
JDS59KL001	*Klebsiella pneumoniae*	RO/BG	429	R	R	R	R	R	R	R	R	R	S	S	R	R	R	R	S	S	R	R	R	S		SHV-11	CTX-M-15				ST 395
JDS59KL002	*Klebsiella pneumoniae*	RO/BG	429	R	R	S	R	R	S	R	R	R	S	S	S	S	S	R	S	S	R	R	R	S		SHV-11	CTX-M-15				ST 1540
JDS59KL019	*Klebsiella pneumoniae*	RO/BG	429	R	R	S	R	R	S	R	R	R	S	S	R	R	R	R	S	S	R	R	S	S	TEM-1	SHV-11	CTX-M-15				ST 976
JDS63EC012	*E*. *coli*	RO	154	R	S	S	R	R	S	R	S	S	S	S	S	S	S	S	S	S	S	S	S	S			CTX-M-1				ST 58
JDS68EB030	*Enterobacter cancerogenus*	RO	104	R	R	R	R	R	R	R	R	S	S	S	S	S	S	S	S	S	S	S	S	S		SHV-2					n.t.
JDS68KL011	*Klebsiella pneumoniae*	RO	104	R	R	S	R	R	S	R	R	S	S	S	R	R	R	R	S	S	S	R	S	S		SHV-1	CTX-M-15				ST 15
JDS68KL012	*Klebsiella pneumoniae*	RO	104	R	R	S	R	R	S	R	R	R	S	S	R	S	R	R	S	S	S	R	S	S	TEM-1	SHV-1	CTX-M-55				ST 15
JDS68KL013	*Klebsiella pneumoniae*	RO	104	R	S	S	R	R	S	R	R	R	S	S	R	R	R	R	S	S	S	R	S	S	TEM-1	SHV-1	CTX-M-55				ST 15
JDS68KL014	*Klebsiella pneumoniae*	RO	104	R	R	S	R	R	S	R	R	R	S	S	R	R	R	R	S	S	S	R	S	S	TEM-1	SHV-1	CTX-M-55				ST 15
JDS68KL015	*Klebsiella pneumoniae*	RO	104	R	S	S	R	R	R	R	R	R	S	S	R	R	R	R	S	S	S	R	S	S	TEM-1	SHV-1	CTX-M-15				ST 15

Resistance pattern, encoded beta-lactamases (TEM, SHV CTX-M-1 group, CTX-M-9 group, KPC and NDM) and MLST (if detectable).

Antibiotic susceptibility is depicted with S for susceptible and R for resistant (highlighted in orange), classes of antibiotics are marked by different colours. Beta lactam antibiotics (red): Ampicillin, AM; amoxicillin/clavulanic acid, AMC; piperacillin/tazobactam, TZP; cefalexin, CN; cefuroxime, CXM; cefoxitin, FOX; cefotaxime, CTX; ceftazidime, CAZ; cefepime, FEP; meropenem, MEM and imipenem, IPM; Quinolones (green) moxifloxacin, MXF; ciprofloxacin, CIP and nalidixic acid, NA. Aminoglycosides (blue): gentamicin, GM and amikacine, AN. Tetracycline antibiotics (yellow): tigecycline, TGC and tetracycline, TE. Other classes (white) trimethoprim/sulfamethoxazole, SXT (inhibition of folic acid synthesis); chloramphenicol, C (chloramphenicol) and colistin, CL (polymyxin).

All isolates were not susceptible to ampicillin and cephalosporins, with the exception of ceftazidime (with 77.14% resistant isolates, including all *Enterobacter* spp. isolates) and cefepime (65.71% resistant isolates). In contrast, the majority of the isolates revealed susceptibility to tazobactam and the cephamycine cefoxitin with only 22.85% and 37.14% resistant isolates.

Co-resistance to sulfamethoxazole/trimethoprim (74.29% of resistant isolates) ciprofloxacin (71.43%), moxifloxacin (68.57%) and gentamicin (62.86%) was very common. Only four of the ten tested non beta-lactam antibiotic displayed less than 50% resistance. Chloramphenicol revealed resistance in 22.86% and amikacine in three (8.57%) isolates, whereas all isolates were susceptible to colistin and tigecycline.

Only five isolates (three *E*. *coli* and two *Enterobacter* spp.) were not classified as multiresistant. Six isolates, including all other *Enterobacter* spp., two *Klebsiella pneumoniae* and one *E*. *coli* revealed resistances at least to one tested antibiotic out of six represented classes.

Analyzing the genetic background of ESBL resistance, the dominant ESBL family was CTX-M, represented by members of the CTX-M-1 (present in 29 isolates) and CTX-M-9 (one isolate) groups. Genes for CTX-M-15 were found in 20 isolates and these were the most common, also the only ESBL present in *E*. *coli* (nine isolates), *Klebsiella pneumoniae* (ten isolates), and *Enterobacter* spp. (one isolate). CTX-M-1 (five isolates) occured in the only *Enterobacter asburiae* isolate and in four *E*. *coli*; CTX-M-3 (two) and CTX-M-27 (one) only occur in *E*. *coli*. On the other hand, CTX-M-55 was detected in three *Klebsiella pneumoniae*, representing one clone isolated at JDS68.

SHV-ESBL was represented by one SHV-2 (JDS68EB030, *Enterobacter cancerogenus*) and five SHV-12 (two *Enterobacter* spp., two *K*. *pneumoniae* and one *E*. *coli*). All *K*. *pneumoniae* without a SHV-12 harbored (as a chromosomal feature of this species) also genes for a non-ESBL variant of SHV (SHV-1 or SHV-11).

The isolates JDS38EB037 (*Enterobacter cloacae*) with TEM-3 was the only isolate with a TEM-ESBL. Non-ESBL TEM-1 was present in 19 isolates.

Multilocus sequence typing (MLST) was performed with all organisms with established MLST protocols in order to be able to compare them more easily to clinical or other environmental isolates.

*E*. *coli* MLST revealed twelve different ST´s, ST10, ST48, ST58, ST69, ST131, ST205, ST405, ST617; ST1914, ST3171, ST5688 and ST5689; *Klebsiella pneumoniae* ST15, ST101,ST395, ST395 and ST2151; and the three *Enterobacter cloacae* revealed ST145, ST159 and ST505 (first reported in this study).

With one exception all detected MLST STs were only present at one sampling site, whereas the *Klebsiella* MLST ST15 was present at three sampling sites (JDS02 (DE), JDS38 (RS) and JDS68 (RO)), including the isolates (JDS02KL027 and JDS38KL007) of *K*. *pneumoniae* that harbored two different ESBL genes (Table 3).

### Detection and characterization of carbapenemase harboring *Enterobacteriacea*

Three of the 35 ESBL harboring *Enterobacteriaceae* were resistant to meropenem and imipenem, and revealed the presence of carbapenemases genes.

JDS38KL007 *Klebsiella pneumoniae* harbored the gene for NDM-1. In this isolate genes for CTX-M-15, SHV-1 and TEM-1 could also be detected. Out of all isolates in this study this was the one which was resistant to most of the tested antibiotics, leaving only two of them, colistin and tigecycline as susceptible. *Klebsiella pneumoniae* MLST resulted in ST101. The second carbapenem resistant *Klebsiella pneumoniae* JDS38KL045 harbored the gene for KPC-2. It also harbored genes encoding CTX-M-15 and a wild type SHV (SHV-11). Susceptibility testing revealed resistance to all tested antibiotics with the exception of colistin, chloramphenicol, tetracycline and tigecycline. Both *Klebsiella pneumoniae* were isolated at the sampling site upstream Pancevo (Serbia).

JDS59EB009 *Enterobacter asburiae* harbored the KPC-2 carbapenemase, alongside with the genes for the other beta-lactamases CTX-M-1, SHV-12 and TEM-1. It was resistant to all tested beta-lactam antibiotics and the other tested antibiotics with the exception of colistin, moxifloxacine amikacin and tigecycline. It was isolated downstream Arges (RO/BG).

## Discussion

*E*. *coli* used to be a handy candidate for treatment with almost every antibiotic. But times have changed and nowadays *E*. *coli* strains seem to have become super bugs. Furthermore, the fully susceptible and easy to treat *E*. *coli* wild type could soon become a minority. This change has already taken place in clinical settings in most European countries, as up to 80% of all isolated *E*. *coli* show already one or more acquired antibiotic resistance. But it has also started to occur in the human community (without direct clinical impact), animals or in (waste) water [5,34–39]. The proportion of resistant *E*. *coli* in the River Danube also reflects this trend with more than 1/3 of all isolates (in total and in all three stretches respectively). Furthermore 10% of the isolates were already multiresistant affecting clinically relevant antibiotics. Comparing these results to other studies (India, China, Nigeria), the proportion of resistant bacteria is lower [39–42]. Unfortunately there are only a few recent European studies on surface waters available. These studies also show lower resistance rates for *E*.*coli* (below 50% for river or waste water) [38,43].

The results of this study show that *E*. *coli* is more affected by the acquisition and stable integration of resistance genes in an aquatic environment than *Klebsiella*. Even if we take into account that ampicillin resistance is intrinsic in *Klebsiella*, this ratio is not changed. Only 23 (3.66°%) of all tested *E*. *coli* revealed exclusive resistance to ampicillin. Hence there are still 1/3 of *E*. *coli* isolates with other acquired resistances remaining. This difference is also supported by other studies, although there are only a few studies which regard the presence of antibiotic resistance proportion in *Klebsiella* in water environment [17]. In general *E*. *coli* is more affected by the spread of resistance (e.g. ESBL) in communities than other *Enterobacteriaceae*. In contrast to this *Klebsiella* spp. or *Enterobacter* spp. are more often multiresistant than *E*. *coli* in clinical settings, especially in intensive care units [44–46].

When comparing neighboring countries to their related River Danube stretches the downstream countries Bulgaria and Rumania have higher resistance rates in clinical isolates of *E*. *coli* and *Klebsiella pneumoniae* than countries from the upper River Danube regions (e.g. aminoglycosides, fluroquinolones, 3^rd^ generation cephalosporins and carbapenems) [5]. But this is only reflected in a lower proportion of *Klebsiella pneumoniae* with wild type susceptibility pattern in the downstream stretch and the more frequent isolation of *Enterobacteriaceae* with resistance to 3^rd^ generation cephalosporins.

Under non-selective culture conditions only three sampling sites revealed ESBL positive *E*. *coli* or *Klebsiella*, representing a proportion of four out of 629 *E*. *coli* and two out of 319 *Klebsiella*, less than 1% of the isolates. There were also only six out of 14 sampling sites with ESBL positive *Enterobacteriaceae*. The presence of ESBL in the first and the last sampling sites confirms the suspected presence of ESBL over the total course of the River Danube.

The isolated genes represent the most dominant ESBL in Europe. CTX-M-15 has spread wildly in hospital and community settings in the last ten years [39]. Well known *E*. *coli* host strains for CTX-M enzymes like ST10, ST69, ST131or ST405 or *Klebsiella pneumoniae* ST15 are present in the Danube water [47–50].

According to the literature the following potential sources, could be assigned to the identified ST-types: ST10 with CTX-M-15 is found in surface water and fish; ST69 with CTX-M-15, ST131 with CTX-M-15 or CTX-M-27, ST405 with CTX-M-15 are found also in surface water, but their primary sources are humans. ST48 with CTX-M-1 is a potential avian pathogen. [51–53]

TEM ESBL was very rare in the Danube isolates. No TEM-52 was detectable; this ESBL is common in human and farm animals but without the dominance of the detected CTX-M and SHV genes. Other studies from Europe report the dominance of CTX-M (including CTX-M-1, CTX-M-3, CTX-M-15, CTX-M-27 and CTX-M-55) and the presence of SHV and the absence of TEM-52 or TEM ESBL in many different surface waters [12,54,55].

Detection of ESBL harboring *Enterobacteriaceae* has become common in surface water (including drinking water) worldwide [12,16]. Recent reports suggest that at the end of this decade it will be the same for *Enterobacteriaceae* producing carbapenemases. The occurrence of KPC, OXA-48 and VIM in waste water, rivers and lakes is already documented for Europe [56,57].

The detection of two KPC-2 producers in the River Danube was not totally unexpected, especially at sampling sites where neighboring countries have to deal with high prevalence of carbapenem resistant *Enterobacteriaceae* in clinical isolates [50,58].

NDM-1 harboring bacteria have been present in the Balkan region since the end of the last decade with several reports from the west and south Balkan. Up to now, these findings were restricted to clinical isolates. A recent study published by Novovic et al. that included also samples from the Danube River collected at the same time as the JDS samples did not find any NDM-1 producer in the environmental water. In our study we were for the first time able to detect this Balkan NDM-1 outside a medical setting [15,59–62]. The detected NDM-1 harboring *Klebsiella pneumoniae* ST101 is also associated with carbapenem resistant *Klebsiella* in the Mediterranean region, but this resistance is mediated via KPC-2 and OXA-48 [50,63].

All three carbapenemase producers and most of the ESBL isolates leave only a few therapeutic options. A few years ago the occurrence of this kind of bacteria resulted in alarming case reports [45]. Now they are present in one of Europe’s biggest rivers and it took only less than 1 liter of surface water to isolate them.

## Conclusion

This study clearly demonstrates the presence of acquired antibiotic resistance in *Enterobacteriaceae*, in one of Europe’s biggest surface water systems caused by human. It is even more alarming as not only a few isolates, detected under selective conditions, are affected, but in some stretches nearly up to 50% of all isolates show altered resistance. Also the low number of ESBL, which could be only found in half of all the sampling sites, is not due to the lack of emergence but more likely caused by the small sample volume, a known study limitation (lack of space on the JDS3 ships).

The River Danube serves as a reservoir for nearly all clinically important antibiotic resistances in *Enterobacteriaceae*. Further studies will have to clarify if the proportion of resistant bacteria has reached a stable level or if wild type susceptibility patterns will be in the minority soon.

## Supporting Information

S1 Table List of all *E*. *coli* isolates and their susceptibility pattern. (XLSX)

S2 Table List of all *Klebsiella* spp. isolates and their susceptibility pattern. (XLSX)

S3 Table River kilometers and geographic coordinates of the sampling sites. (XLSX)

## References

[1] Viens AM, Littmann J. Is Antimicrobial Resistance a Slowly Emerging Disaster? Public Health Ethics. 2015;8: 255–265. 10.1093/phe/phv015 26566396 PMC4638061

[2] Livermore DM. Fourteen years in resistance. Int J Antimicrob Agents. 2012;39: 283–294. 10.1016/j.ijantimicag.2011.12.012 22386741

[3] Iredell J, Brown J, Tagg K. Antibiotic resistance in Enterobacteriaceae: mechanisms and clinical implications. BMJ. 2016;352: h6420 10.1136/bmj.h6420 26858245

[4] Taylor NG, Verner-Jeffreys DW, Baker-Austin C. Aquatic systems: maintaining, mixing and mobilising antimicrobial resistance? Trends Ecol Evol. 2011;26: 278–284. 10.1016/j.tree.2011.03.004 21458879

[5] ECDC. European Centre for Disease Prevention and Control (2014). Antimicrobial resistance surveillance in Europe 2013. Annual Report of the European Antimicrobial Resistance Surveilance Network (EARS-Net). 2014.

[6] Kummerer K. Antibiotics in the aquatic environment—a review—part I. Chemosphere. 2009;75: 417–434. 10.1016/j.chemosphere.2008.11.086 19185900

[7] Kummerer K. Antibiotics in the aquatic environment—a review—part II. Chemosphere. 2009;75: 435–441. 10.1016/j.chemosphere.2008.12.006 19178931

[8] Bouki C, Venieri D, Diamadopoulos E. Detection and fate of antibiotic resistant bacteria in wastewater treatment plants: a review. Ecotoxicol Environ Saf. 2013;91: 1–9. 10.1016/j.ecoenv.2013.01.016 23414720

[9] Baker-Austin C, Wright MS, Stepanauskas R, McArthur JV. Co-selection of antibiotic and metal resistance. Trends Microbiol. 2006;14: 176–182. 10.1016/j.tim.2006.02.006 16537105

[10] Davison J. Genetic exchange between bacteria in the environment. Plasmid. 1999;42: 73–91. 10.1006/plas.1999.1421 10489325

[11] Rizzo L, Manaia C, Merlin C, Schwartz T, Dagot C, Ploy MC, et al Urban wastewater treatment plants as hotspots for antibiotic resistant bacteria and genes spread into the environment: a review. Sci Total Environ. 2013;447: 345–360. 10.1016/j.scitotenv.2013.01.032 23396083

[12] Zurfluh K, Hachler H, Nuesch-Inderbinen M, Stephan R. Characteristics of extended-spectrum beta-lactamase- and carbapenemase-producing Enterobacteriaceae Isolates from rivers and lakes in Switzerland. Appl Environ Microbiol. 2013;79: 3021–3026. 10.1128/AEM.00054-13 23455339 PMC3623138

[13] Schaefer AM, Goldstein JD, Reif JS, Fair PA, Bossart GD. Antibiotic-resistant organisms cultured from Atlantic bottlenose dolphins (Tursiops truncatus) inhabiting estuarine waters of Charleston, SC and Indian River Lagoon, FL. Ecohealth. 2009;6: 33–41. 10.1007/s10393-009-0221-5 19415386

[14] Belmonte O, Drouet D, Alba J, Moiton MP, Kuli B, Lugagne-Delpon N, et al Evolution of Enterobacteriaceae resistance to antibiotics in Reunion Island: emergence of extended-spectrum beta-lactamases. Pathol Biol (Paris). 2010;58: 18–24. 19864085 10.1016/j.patbio.2009.07.021

[15] Novovic K, Filipic B, Veljovic K, Begovic J, Mirkovic N, Jovcic B. Environmental waters and blaNDM-1 in Belgrade, Serbia: endemicity questioned. Sci Total Environ. 2015;511: 393–398. 10.1016/j.scitotenv.2014.12.072 25569574

[16] Zhang H, Zhou Y, Guo S, Chang W. Prevalence and characteristics of extended-spectrum beta-lactamase (ESBL)-producing Enterobacteriaceae isolated from rural well water in Taian, China, 2014. Environ Sci Pollut Res Int. 2015;22: 11488–11492. 10.1007/s11356-015-4387-9 25821088

[17] Livermore DM, Canton R, Gniadkowski M, Nordmann P, Rossolini GM, Arlet G, et al CTX-M: changing the face of ESBLs in Europe. J Antimicrob Chemother. 2007;59: 165–174. 10.1093/jac/dkl483 17158117

[18] Carattoli A. Animal reservoirs for extended spectrum beta-lactamase producers. 2008;14: 117–123. 10.1111/j.1469-0691.2007.01851.x 18154535

[19] Torneke K, Torren-Edo J, Grave K, Mackay DK. The management of risk arising from the use of antimicrobial agents in veterinary medicine in EU/EEA countries—a review. J Vet Pharmacol Ther. 2015;38: 519–528. 10.1111/jvp.12226 25855219

[20] Rahube TO, Yost CK. Characterization of a mobile and multiple resistance plasmid isolated from swine manure and its detection in soil after manure application. J Appl Microbiol. 2012;112: 1123–1133. 10.1111/j.1365-2672.2012.05301.x 22486928

[21] JDS 3. Joint Danube Survey 3 A Comprehensive Analysis of Danube Water Quality. Vienna: ICPDR—International Commission for the Protection of the Danube River; 2015.

[22] EUCAST. European Committee on Antimicrobial Susceptibility Testing (EUCAST). 2013. Available: http://www.eucast.org;.

[23] CLSI, Clinical and Laboratory Standards Institute. Performance Standards for antimicrobial susceptibility testing: 18th informational supplement CLSI document M100-S18. 2008;Clinical and Laboratory Standards Institute, Wayne, PA.

[24] Gales AC, Reis AO, Jones RN. Contemporary assessment of antimicrobial susceptibility testing methods for polymyxin B and colistin: review of available interpretative criteria and quality control guidelines. J Clin Microbiol. 2001;39: 183–190. 10.1128/JCM.39.1.183-190.2001 11136768 PMC87699

[25] Boyen F, Vangroenweghe F, Butaye P, De Graef E, Castryck F, Heylen P, et al Disk prediffusion is a reliable method for testing colistin susceptibility in porcine E. coli strains. Vet Microbiol. 2010;144: 359–362. 10.1016/j.vetmic.2010.01.010 20172663

[26] Lee K, Chong Y, Shin HB, Kim YA, Yong D, Yum JH. Modified Hodge and EDTA-disk synergy tests to screen metallo-beta-lactamase-producing strains of Pseudomonas and Acinetobacter species. Clin Microbiol Infect. 2001;7: 88–91. 11298149 10.1046/j.1469-0691.2001.00204.x

[27] Pitout JD, Reisbig MD, Venter EC, Church DL, Hanson ND. Modification of the double-disk test for detection of enterobacteriaceae producing extended-spectrum and AmpC beta-lactamases. J Clin Microbiol. 2003;41: 3933–3935. 10.1128/JCM.41.8.3933-3935.2003 12904422 PMC179840

[28] Kiratisin P, Apisarnthanarak A, Laesripa C, Saifon P. Molecular characterization and epidemiology of extended-spectrum-beta-lactamase-producing Escherichia coli and Klebsiella pneumoniae isolates causing health care-associated infection in Thailand, where the CTX-M family is endemic. Antimicrob Agents Chemother. 2008;52: 2818–2824. 10.1128/AAC.00171-08 18505851 PMC2493136

[29] Eckert C, Gautier V, Saladin-Allard M, Hidri N, Verdet C, Ould-Hocine Z, et al Dissemination of CTX-M-type beta-lactamases among clinical isolates of Enterobacteriaceae in Paris, France. Antimicrob Agents Chemother. 2004;48: 1249–1255. 10.1128/AAC.48.4.1249-1255.2004 15047527 PMC375249

[30] Bradford PA, Bratu S, Urban C, Visalli M, Mariano N, Landman D, et al Emergence of carbapenem-resistant Klebsiella species possessing the class A carbapenem-hydrolyzing KPC-2 and inhibitor-resistant TEM-30 beta-lactamases in New York City. Clin Infect Dis. 2004;39: 55–60. 10.1086/421495 15206053

[31] Jolley KA, Maiden MC. BIGSdb: Scalable analysis of bacterial genome variation at the population level. BMC Bioinformatics. 2010;11: 595-2105-11-595. 10.1186/1471-2105-11-595 PMC3004885 21143983

[32] Diancourt L, Passet V, Verhoef J, Grimont PA, Brisse S. Multilocus sequence typing of Klebsiella pneumoniae nosocomial isolates. J Clin Microbiol. 2005;43: 4178–4182. 10.1128/JCM.43.8.4178-4182.2005 16081970 PMC1233940

[33] Brisse S, Fevre C, Passet V, Issenhuth-Jeanjean S, Tournebize R, Diancourt L, et al Virulent clones of Klebsiella pneumoniae: identification and evolutionary scenario based on genomic and phenotypic characterization. PLoS One. 2009;4: e4982 10.1371/journal.pone.0004982 19319196 PMC2656620

[34] Paterson DL. Resistance in gram-negative bacteria: enterobacteriaceae. Am J Med. 2006;119: S20–8; discussion S62-70. 10.1016/j.amjmed.2006.03.013 16735147

[35] Luczkiewicz A, Jankowska K, Fudala-Ksiazek S, Olanczuk-Neyman K. Antimicrobial resistance of fecal indicators in municipal wastewater treatment plant. Water Res. 2010;44: 5089–5097. 10.1016/j.watres.2010.08.007 20810144

[36] Pignato S, Coniglio MA, Faro G, Lefevre M, Weill FX, Giammanco G. Molecular epidemiology of ampicillin resistance in Salmonella spp. and Escherichia coli from wastewater and clinical specimens. Foodborne Pathog Dis. 2010;7: 945–951. 10.1089/fpd.2009.0504 20367333

[37] Ewers C, Bethe A, Semmler T, Guenther S, Wieler LH. Extended-spectrum beta-lactamase-producing and AmpC-producing Escherichia coli from livestock and companion animals, and their putative impact on public health: a global perspective. Clin Microbiol Infect. 2012;18: 646–655. 10.1111/j.1469-0691.2012.03850.x 22519858

[38] Reinthaler FF, Galler H, Feierl G, Haas D, Leitner E, Mascher F, et al Resistance patterns of Escherichia coli isolated from sewage sludge in comparison with those isolated from human patients in 2000 and 2009. J Water Health. 2013;11: 13–20. 10.2166/wh.2012.207 23428545

[39] Poonia S, Singh TS, Tsering DC. Antibiotic susceptibility profile of bacteria isolated from natural sources of water from rural areas of East sikkim. Indian J Community Med. 2014;39: 156–160. 10.4103/0970-0218.137152 25136156 PMC4134531

[40] Titilawo Y, Sibanda T, Obi L, Okoh A. Multiple antibiotic resistance indexing of Escherichia coli to identify high-risk sources of faecal contamination of water. Environ Sci Pollut Res Int. 2015;22: 10969–10980. 10.1007/s11356-014-3887-3 25779106

[41] Zheng S, Qiu X, Chen B, Yu X, Liu Z, Zhong G, et al Antibiotics pollution in Jiulong River estuary: source, distribution and bacterial resistance. Chemosphere. 2011;84: 1677–1685. 10.1016/j.chemosphere.2011.04.076 21620433

[42] Zhang X, Li Y, Liu B, Wang J, Feng C, Gao M, et al Prevalence of veterinary antibiotics and antibiotic-resistant Escherichia coli in the surface water of a livestock production region in northern China. PLoS One. 2014;9: e111026 10.1371/journal.pone.0111026 25372873 PMC4220964

[43] Blaak H, Lynch G, Italiaander R, Hamidjaja RA, Schets FM, de Roda Husman AM. Multidrug-Resistant and Extended Spectrum Beta-Lactamase-Producing Escherichia coli in Dutch Surface Water and Wastewater. PLoS One. 2015;10: e0127752 10.1371/journal.pone.0127752 26030904 PMC4452230

[44] Rice LB. The clinical consequences of antimicrobial resistance. Curr Opin Microbiol. 2009;12: 476–481. 10.1016/j.mib.2009.08.001 19716760

[45] Nordmann P, Dortet L, Poirel L. Carbapenem resistance in Enterobacteriaceae: here is the storm! Trends Mol Med. 2012;18: 263–272. 10.1016/j.molmed.2012.03.003 22480775

[46] Korzeniewska E, Harnisz M. Extended-spectrum beta-lactamase (ESBL)-positive Enterobacteriaceae in municipal sewage and their emission to the environment. J Environ Manage. 2013;128: 904–911. 10.1016/j.jenvman.2013.06.051 23886578

[47] Mathers AJ, Peirano G, Pitout JD. Escherichia coli ST131: The quintessential example of an international multiresistant high-risk clone. Adv Appl Microbiol. 2015;90: 109–154. 10.1016/bs.aambs.2014.09.002 25596031

[48] Pietsch M, Eller C, Wendt C, Holfelder M, Falgenhauer L, Fruth A, et al Molecular characterisation of extended-spectrum beta-lactamase (ESBL)-producing Escherichia coli isolates from hospital and ambulatory patients in Germany. Vet Microbiol. 2015. 10.1016/j.vetmic.2015.11.028 26654217

[49] Reuland EA, Overdevest IT, Al Naiemi N, Kalpoe JS, Rijnsburger MC, Raadsen SA, et al High prevalence of ESBL-producing Enterobacteriaceae carriage in Dutch community patients with gastrointestinal complaints. Clin Microbiol Infect. 2013;19: 542–549. 10.1111/j.1469-0691.2012.03947.x 22757622

[50] Markovska R, Stoeva T, Schneider I, Boyanova L, Popova V, Dacheva D, et al Clonal dissemination of multilocus sequence type ST15 KPC-2-producing Klebsiella pneumoniae in Bulgaria. APMIS. 2015;123: 887–894. 10.1111/apm.12433 26303718

[51] Dissanayake DR, Octavia S, Lan R. Population structure and virulence content of avian pathogenic Escherichia coli isolated from outbreaks in Sri Lanka. Vet Microbiol. 2014;168: 403–412. 10.1016/j.vetmic.2013.11.028 24388626

[52] Sola-Gines M, Gonzalez-Lopez JJ, Cameron-Veas K, Piedra-Carrasco N, Cerda-Cuellar M, Migura-Garcia L. Houseflies (Musca domestica) as Vectors for Extended-Spectrum beta-Lactamase-Producing Escherichia coli on Spanish Broiler Farms. Appl Environ Microbiol. 2015;81: 3604–3611. 10.1128/AEM.04252-14 25795670 PMC4421041

[53] Muller A, Stephan R, Nuesch-Inderbinen M. Distribution of virulence factors in ESBL-producing Escherichia coli isolated from the environment, livestock, food and humans. Sci Total Environ. 2016;541: 667–672. 10.1016/j.scitotenv.2015.09.135 26437344

[54] Ojer-Usoz E, Gonzalez D, Garcia-Jalon I, Vitas AI. High dissemination of extended-spectrum beta-lactamase-producing Enterobacteriaceae in effluents from wastewater treatment plants. Water Res. 2014;56: 37–47. 10.1016/j.watres.2014.02.041 24651016

[55] Zarfel G, Galler H, Feierl G, Haas D, Kittinger C, Leitner E, et al Comparison of extended-spectrum-beta-lactamase (ESBL) carrying Escherichia coli from sewage sludge and human urinary tract infection. Environ Pollut. 2013;173: 192–199. 10.1016/j.envpol.2012.09.019 23202650

[56] Poirel L, Barbosa-Vasconcelos A, Simoes RR, Da Costa PM, Liu W, Nordmann P. Environmental KPC-producing Escherichia coli isolates in Portugal. Antimicrob Agents Chemother. 2012;56: 1662–1663. 10.1128/AAC.05850-11 22203588 PMC3294942

[57] Galler H, Feierl G, Petternel C, Reinthaler FF, Haas D, Grisold AJ, et al KPC-2 and OXA-48 carbapenemase-harbouring Enterobacteriaceae detected in an Austrian wastewater treatment plant. Clin Microbiol Infect. 2013. 10.1111/1469-0691.12336 24033741

[58] Toth A, Damjanova I, Puskas E, Janvari L, Farkas M, Dobak A, et al Emergence of a colistin-resistant KPC-2-producing Klebsiella pneumoniae ST258 clone in Hungary. Eur J Clin Microbiol Infect Dis. 2010;29: 765–769. 10.1007/s10096-010-0921-3 20401676

[59] Zarfel G, Hoenigl M, Wurstl B, Leitner E, Salzer HJ, Valentin T, et al Emergence of carbapenem-resistant Enterobacteriaceae in Austria, 2001–2010. Clin Microbiol Infect. 2011;17: E5–8. 10.1111/j.1469-0691.2011.03659.x 21939472

[60] Mazzariol A, Bosnjak Z, Ballarini P, Budimir A, Bedenic B, Kalenic S, et al NDM-1-producing Klebsiella pneumoniae, Croatia. Emerg Infect Dis. 2012;18: 532–534. 10.3201/eid1803.1103890 22377049 PMC3309569

[61] Hammerum AM, Toleman MA, Hansen F, Kristensen B, Lester CH, Walsh TR, et al Global spread of New Delhi metallo-beta-lactamase 1. Lancet Infect Dis. 2010;10: 829–830. 10.1016/S1473-3099(10)70276-0 21109169

[62] Voulgari E, Gartzonika C, Vrioni G, Politi L, Priavali E, Levidiotou-Stefanou S, et al The Balkan region: NDM-1-producing Klebsiella pneumoniae ST11 clonal strain causing outbreaks in Greece. J Antimicrob Chemother. 2014;69: 2091–2097. 10.1093/jac/dku105 24739146

[63] Del Franco M, Paone L, Novati R, Giacomazzi CG, Bagattini M, Galotto C, et al Molecular epidemiology of carbapenem resistant Enterobacteriaceae in Valle d'Aosta region, Italy, shows the emergence of KPC-2 producing Klebsiella pneumoniae clonal complex 101 (ST101 and ST1789). BMC Microbiol. 2015;15: 260-015-0597-z. 10.1186/s12866-015-0597-z PMC4640108 26552763

